# Posture-Induced Intraocular Pressure Changes after iStent Inject W Combined with Phacoemulsification in Open Angle Glaucoma Patients

**DOI:** 10.3390/medicina59030423

**Published:** 2023-02-21

**Authors:** Kentaro Iwasaki, Shogo Arimura, Yusuke Orii, Masaru Inatani

**Affiliations:** Department of Ophthalmology, Faculty of Medical Sciences, University of Fukui, Fukui 910-1193, Japan

**Keywords:** iStent inject W, phacoemulsification, posture-induced IOP change, open-angle glaucoma, surgical efficacy

## Abstract

*Background and Objectives*: The purpose of this study was to evaluate the posture-induced intraocular pressure (IOP) changes after iStent inject W combined with phacoemulsification procedure in Japanese patients with open-angle glaucoma. *Materials and Methods*: We prospectively evaluated the posture-induced IOP changes after surgery. The primary outcome was the posture-induced IOP changes postoperatively. Secondary outcome measures included postoperative complications, visual acuity, visual field, and corneal endothelial cell density. *Results*: This study completed the prospective observation for 15 eyes (15 patients). The mean preoperative IOP with the Goldmann applanation tonometer was 16.0 ± 2.6 mm Hg with a mean glaucoma medication usage of 2.5 ± 1.2, which decreased to 14.4 ± 2.4 mm Hg (*p* = 0.14) and 0.5 ± 0.9 medications (*p* < 0.01), respectively, 12 months postoperatively. The mean baseline IOP with the ICare was 12.0 ± 2.7 mmHg in the sitting position, which significantly increased to 15.2 ± 3.8 mmHg in the lateral decubitus position (*p* < 0.01). This postural IOP difference was 3.2 ± 2.2 mmHg and 3.2 ± 2.4 mmHg at baseline and 12 months postoperatively, respectively, with no significant changes (*p* > 0.99). *Conclusions*: iStent inject W combined with cataract surgery reduced the IOP and the number of glaucoma medications during short-term follow-ups with high safety. However, iStent inject W did not affect the degree of posture-induced IOP changes.

## 1. Introduction

Glaucoma is a group of neurodegenerative diseases resulting in the loss of retinal ganglion cells. Intraocular pressure (IOP) plays a key role in glaucoma progression. IOP variations occur because of altering physiological conditions, including age, diurnal and seasonal cycles, and postural alternations, especially at sleeping time [1,2,3]. Controlling all of these variables to get an accurate IOP value is difficult. Additionally, all of these factors make the determination of the effect of the IOP on glaucoma progression difficult. Of these factors, postural changes have a close association with functional and morphological disorders in glaucoma [4,5,6,7]. It has been reported that the postural response of IOP does not change after the use of glaucoma medications (timolol maleate, latanoprost, and brinzolamide) [8] and the treatment of argon laser trabeculoplasty [9].

Glaucoma filtering surgery is the most effective method of achieving a lower IOP for patients with glaucoma with medically uncontrollable IOP [10]. Trabeculectomy is the most common surgery among filtering surgeries in the world. Several studies investigated posture-induced IOP changes after filtering surgery and suggested that the trabeculectomy reduced the effect on the degree of posture-induced IOP changes and measuring posture-induced IOP changes might be a method for assessing whether the bleb has successful filtration [11,12,13]. However, patients performed with trabeculectomy often encounter postoperative visual impairment. Hypotonic maculopathy, massive postoperative hyphema, cataract progression, and fixation loss or wipeout decrease visual acuity after trabeculectomy. These complications are a serious problem of trabeculectomy.

Conversely, recently, newer techniques performed using an ab interno approach to the trabecular meshwork are becoming popular because of their minimal invasion and fewer complications, and the range of surgical options available to treat glaucoma has significantly increased [14,15,16]. The trabecular meshwork and inner walls of Schlemm’s canal are the main sites of resistance to aqueous outflow [17,18,19]. The iStent, Kahook Dual Blade, the Trabectome, TrabEx+, Microhook, and the Suture trabeculotomy are approved for use in Japan as micro-invasive glaucoma surgeries (MIGS), and their procedures provide more safety and shorter recovery periods than other glaucoma surgeries. The iStent inject W (Glaukos Corporation, Laguna Hills, CA, USA) is a bypass implant between the anterior chamber and Schlemm’s canal to decrease aqueous outflow resistance among MIGS [20,21]. Several studies have suggested the IOP lowering effect of dual iStent inject device implantation in mild-to-moderate open-angle glaucoma [22,23,24,25]. However, posture-induced IOP changes after the MIGS procedures, including iStent inject W, have not been evaluated. Therefore, the current study aims to evaluate the posture-induced IOP changes after iStent inject W combined with phacoemulsification.

## 2. Materials and Methods

### 2.1. Patient Selection

This prospective observational clinical cohort study was approved by the institutional review board of the Fukui University Hospital (Fukui, Japan). Our study protocol adhered to the tenets of the Declaration of Helsinki. We obtained written informed consent for this surgery, but the informed consent requirement for this study was waived because of the observational study.

This prospective study evaluated the posture-induced IOP changes after iStent inject W (Glaukos Corporation, Laguna Hills, CA, USA) combined with phacoemulsification. We recruited patients from January 2021 to September 2021 at the Fukui University Hospital. The inclusion criteria were being aged ≥20 years and having open-angle glaucoma (primary open-angle or exfoliation) without a history of intraocular surgery. We followed the criteria for usage requirements of iStent inject W from the Japanese Ophthalmological Society. The exclusion criteria were patients with visual field mean deviation (Humphrey 24–2, Humphrey Field Analyzer, Humphrey Instruments, San Leandro, CA, USA) <−12.0 dB, patients with a history of intraocular surgery and laser treatment of glaucoma (laser trabeculoplasty, laser iridotomy, and laser gonioplasty), and patients with primary angle-closure glaucoma, secondary glaucoma (except for exfoliation glaucoma), neovascular glaucoma, and congenital glaucoma.

### 2.2. iStent Inject W Device and Surgical Procedures

The iStent inject W device is a new version of the second-generation iStent inject [21]. The device is a 360-µm long stent made of biocompatible heparin-coated titanium. The stent is designed to be implanted through the trabecular meshwork (TM) so that its head lies within Schlemm’s canal (SC) while its 360-µm wide flange remains within the anterior chamber. Its inlet and central canal have an 80-µm inner cross-section, and four 50-µm orifices in the head of the device allow for aqueous outflow from the anterior chamber. The device is intended for ab interno implantation and comes with a preloaded injector containing two devices.

All surgeries were performed by experienced glaucoma specialists. Preoperative medications were to include 0.3% gatifloxacin 3 times per day for 3 days preoperatively. We used topical anesthesia for this surgery. Before the iStent implantation, standard phacoemulsification was performed with intraocular lens implantation through a clear 2.4-mm temporal corneal incision. To visualize a good view of the TM in the nasal angle with a gonioprism (Ocular Hill Open Access Surgical Gonio, Ocular Instruments, Bellevue, WA, USA), the patient’s head and the microscope were tilted by approximately 30 degrees each. The surgeon then filled the anterior chamber with additional viscoelastic material (1% sodium hyaluronate, SEIKAGAKU, Santen Pharmaceutical, Osaka, Japan) to deepen the angle and maintain the anterior chamber depth. The surgeons inserted the iStent preloaded injector into the anterior chamber through the existing corneal incision to the nasal TM. The first stent was implanted into the SC through the TM, and then the second stent was implanted laterally at approximately 60 degrees apart from the first stent. The surgeons removed the viscoelastic material and filled the anterior chamber with a balanced saline solution as needed to achieve physiologic pressure after these procedures. Lastly, the surgeons ensured proper sealing of the corneal incision.

All patients received similar postoperative topical medications, namely, 0.3% gatifloxacin 3 times per day for 1–2 weeks, 0.1% betamethasone phosphate 3 times per day for 3–4 weeks, and 0.1% nepafenac 3 times per day for 2–3 months. Glaucoma medications were stopped upon surgery and resumed according to the surgeon’s discretion at postoperative follow-up visits.

### 2.3. Data Collection

Patient data, including sex, age, glaucoma type, best corrected visual acuity (BCVA), preoperative IOP, postoperative IOP, number of glaucoma medications, visual field mean deviation, central corneal endothelial cell density (ECD), and presence of postoperative complications, were collected. The logarithm of the reciprocal of the decimal BCVA was used to approximate the logarithm of the minimal angle of resolution (LogMAR). The first study-related visit was scheduled 1 week postoperatively; thereafter, follow-up visits occurred 1, 3, 6, and 12 months postoperatively. We assessed the IOP, the number of glaucoma medications, BCVA, mean visual field deviation, and central corneal ECD before surgery and at defined follow-up time points. Additionally, complications were assessed at all follow-up visits. The IOP was measured with a Goldmann applanation tonometer (GAT; AT900, Haag Streit, Koniz, Switzerland) and ICare rebound tonometer (ICare; Tiolat Oy, Helsinki, Finland). The GAT measurement was performed using one eye drop of local anesthetic (Oxybuprocain + Fluorescein). IOP measurement was performed by a single examiner throughout the experimental period. The IOP was first measured in a sitting position with the ICare to determine posture-induced IOP change. Then, the patient was instructed to lie on the bed, turn to the lateral decubitus position, and place the head on the pillow. The body was positioned so that the eye scheduled for the surgery was positioned directly above the fellow eye. The body position was maintained for 5 min, and the IOP was measured in this position with the ICare [7,13]. The IOP was measured by touching the transducer to the center of the patient’s cornea. Three sets of measurements were performed consecutively, with 6 measurements in each set. Means for each set were automatically created, and the mean values were used for the analysis. After the IOP measurement in two positions with the ICare, the GAT measurement was performed in a sitting position. The corneal endothelium was quantified using a noncontact-type specular microscope (Konan Specular Microscope XI FA-3709P; Konan Medical Inc., Hyogo, Japan). The purpose of the study was masked to the examiner. Postoperative complications included hyphema, which was defined as blood niveau formation in the anterior chamber, and an IOP spike defined as an increased IOP of >10 mmHg above baseline within 1 month postoperatively.

### 2.4. Outcome Measures

The primary outcome measures were the posture-induced IOP changes and the number of glaucoma medications used after iStent inject W combined with phacoemulsification. We evaluated the time course of the changes in the IOPs measured with GAT, the number of glaucoma medications used and the IOPs measured in the sitting and lateral decubitus position with the ICare. Secondary outcome measures included postoperative complications, visual acuity, visual field, and corneal ECD. We evaluated the postoperative complications up to 12 months after surgery. Visual acuity, visual field, and corneal ECD were compared at baseline and 12 months after surgery.

### 2.5. Statistical Analysis

We performed univariate comparisons between groups using paired *t*-tests with Bonferroni correction. The longitudinal repeated measures were analyzed using a one-way repeated measures analysis of variance. *P*-values of <0.05 were considered statistically significant. SPSS software version 24.0 (IBM Institute, Inc. Chicago, IL, USA) was used for the statistical analysis.

## 3. Results

### 3.1. Patient Demographic and Clinical Characteristics

This study enrolled a total of 15 eyes (15 patients). All patients were Japanese. The mean age was 73.1 ± 6.3 years, and four were male. Diagnoses consisted of Primary open-angle glaucoma (*n* = 12) and Exfoliation glaucoma (*n* = 3). The mean preoperative IOP was 16.0 ± 2.6 mmHg on a mean of 2.5 ± 1.3 glaucoma medications. The preoperative visual field’s mean deviation was −9.8 ± 4.6 dB. Table 1 summarizes the demographics and baseline clinical characteristics of the patients. All patients completed our protocol.

### 3.2. Primary Outcome

**Figure 1:**
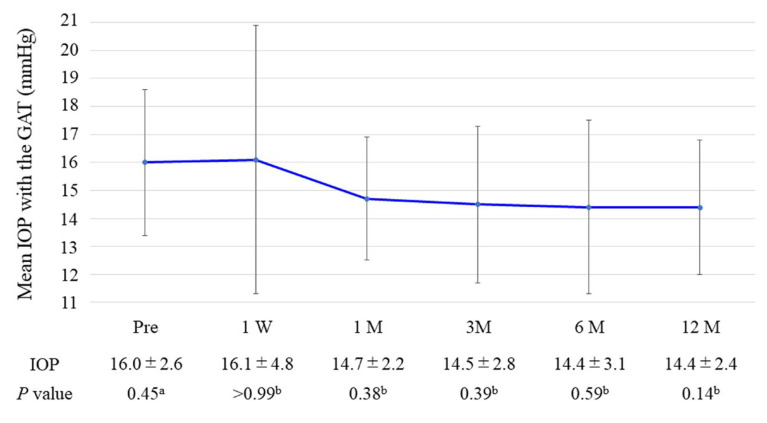
Changes in mean IOP were measured with GAT throughout follow-up. The error bar shows the standard deviation. GAT, Goldmann applanation tonometer; IOP, intraocular pressure; W, week; M, month. *P*-values: ^a^ one-way repeated measures ANOVA; ^b^ paired *t*-test versus baseline with Bonferroni correction.

**Figure 2:**
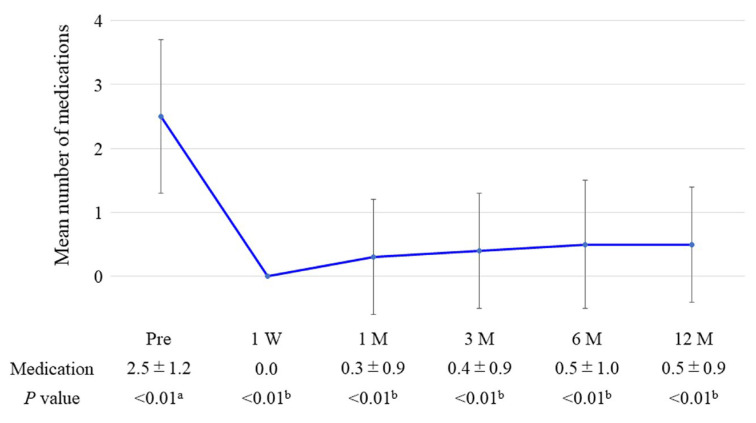
Changes in the mean number of glaucoma medications throughout follow-up. The error bar shows the standard deviation. W, week; M, month. *P*-values: ^a^ one-way repeated measures ANOVA; ^b^ paired *t*-test versus baseline with Bonferroni correction.

The time course of the changes in the IOPs measured with GAT and the number of glaucoma medications used are shown in Figure 1 and Figure 2, respectively, at follow-up time points. The mean preoperative IOP was 16.0 ± 2.6 mm Hg with a mean use of 2.5 ± 1.2 glaucoma medications, and these values were decreased to 14.4 ± 2.4 mm Hg (*p* = 0.14) and 0.5 ± 0.9 medications (*p* < 0.01), respectively, 12 months postoperatively. The medication-free rate overall was 73.3% at 12 months postoperatively. The using ≥1 fewer medications rate from baseline at 12 months postoperatively was 93.3%.

**Figure 3:**
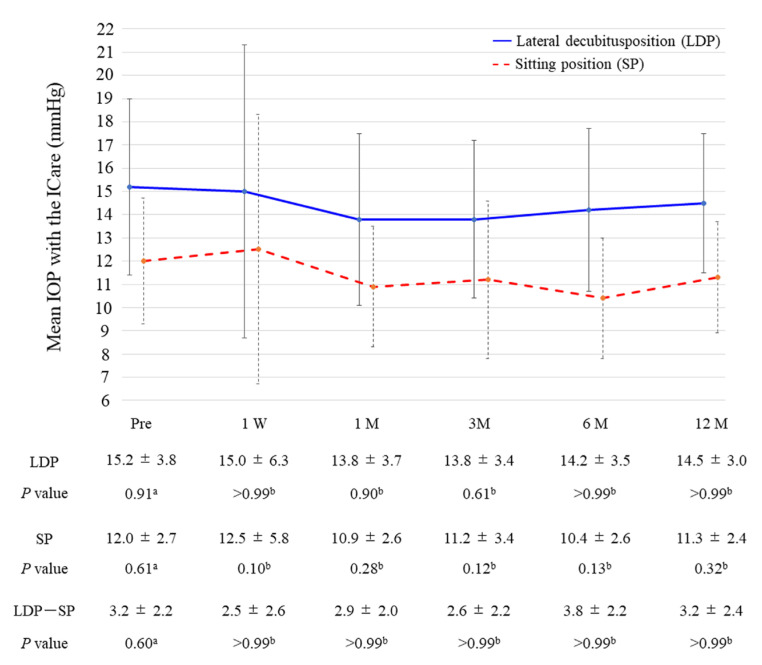
Changes in mean IOP were measured in the sitting and lateral decubitus positions with the ICare throughout follow-up. The error bar shows the standard deviation. IOP, intraocular pressure; W, week; M, month. *P*-values: ^a^ one-way repeated measures ANOVA; ^b^ paired *t*-test versus baseline with Bonferroni correction.

The time course of the IOP changes measured in the sitting and lateral decubitus position with the ICare is shown in Figure 3. The mean baseline IOP with the ICare was 12.0 ± 2.7 mmHg in the sitting position, and the IOP significantly increased to 15.2 ± 3.8 mmHg in the lateral decubitus position (*p* < 0.01). The IOP reduced to 11.3 ± 2.4 mmHg and 14.5 ± 3.0 mmHg in the sitting and lateral decubitus positions 12 months postoperatively, although with no significant differences compared with the baseline. The difference in the IOP between the sitting and lateral decubitus position was 3.2 ± 2.2 mmHg and 3.2 ± 2.4 mmHg at baseline and 12 months postoperatively, with no significant changes (*p* > 0.99).

### 3.3. Secondary Outcomes

Table 2 summarizes the postoperative complications. Hyphema and IOP spikes have not occurred in any cases. One of two iStent was occluded by the iris in two cases (13.3%). These eyes acquired IOP reduction from baseline, so additional intervention to resolve the iStent occlusion was not required. Additional glaucoma surgery for IOP control was not required in any cases at 12 months postoperatively. Cataract surgery-related complications were not observed.

The mean central corneal ECD was 2525 ± 321 cells/mm^2^ at the preoperative, which decreased to 2470 ± 379 cells/mm^2^ at 12 months postoperatively (2.3% reduction; *p* = 0.24). The visual acuity (LogMAR) at baseline was 0.45 ± 0.3, which significantly improved to 0.12 ± 0.2 at 12 months postoperatively (*p* < 0.01), consistent with expectations for cataract surgery. The average visual field MD improved from −9.8 ± 4.6 dB preoperatively to −8.6 ± 3.9 dB at 12 months postoperatively (*p* = 0.091).

## 4. Discussion

Our study aimed to evaluate the posture-induced IOP changes after iStent inject W combined with phacoemulsification. The mean IOP with GAT decreased from 16.0 ± 2.6 mmHg preoperatively to 14.4 ± 2.4 mmHg (*p* = 0.14) at 12 months postoperatively, and the mean number of glaucoma medications from 2.5 ± 1.2 to 0.5 ± 0.9 (*p* < 0.01). The mean baseline IOP with the ICare was 12.0 ± 2.7 mmHg in the sitting position, which significantly increased to 15.2 ± 3.8 mmHg in the lateral decubitus position (*p* < 0.01). This postural IOP difference was 3.2 ± 2.2 mmHg and 3.2 ± 2.4 mmHg at baseline and 12 months postoperatively. No significant postoperative changes were found in postural IOP over time (*p* > 0.99).

Several studies reported on the degree of posture-induced IOP changes after trabeculectomy [11,12,13]. In addition, it has been reported that the use of glaucoma medications and the treatment of argon laser trabeculoplasty has no effect on the degree of posture-induced IOP changes [8,9]. However, posture-induced IOP changes after the MIGS procedures have not been evaluated. Recently, MIGS procedures are becoming popular as primary glaucoma surgery in the world. Therefore, it is important for us to confirm the different surgical outcomes, including posture-induced IOP changes in MIGS procedures. Our present study is the first report about the evaluation of posture-induced IOP changes after iStent inject W combined with phacoemulsification. Furthermore, surgical outcomes of iStent inject W, which is a new version of the second-generation iStent inject, were not reported.

In recent years, several studies of iStent inject combined with cataract surgery have been published. These previous reports showed IOP reductions between 8.7% and 29.4% 12 months postoperatively, with a reduction in medications of 56.0%–94.7% [23,24,26,27,28]. Our surgical outcomes regarding IOP (10.0%) and medication (80.0%) reduction are consistent with those previous reports. This result suggested that iStent inject W combined with cataract surgery achieved IOP and glaucoma medication reductions 12 months postoperatively in Japanese patients with open-angle glaucoma.

ICare is a useful device that can measure IOP regardless of body position. In this study, IOP was easily measured in two body positions using ICare. ICare IOP values were lower by 3.1–4.0 mmHg than GAT values throughout follow-up points in our study. Several previous reports showed that ICare IOP values were lower by 0.4–3.0 mmHg than GAT values [29,30,31,32]. Our result is largely consistent with those previous reports. However, other earlier studies reported that the ICare IOP values were higher by 0.5–2.0 mmHg than the GAT values [33,34,35,36,37,38]. These inconsistent results in different studies may stem from interobserver variability with the GAT IOP measurements [39], manipulation of eyelids by ophthalmologists [40], use of prostaglandins [41], and the age of the study population. Furthermore, the mean IOP reductions after 12 months postoperatively were 1.6 mmHg and 0.7 mmHg with GAT and ICare measurements, respectively, in this study. This difference result of IOP reduction between the two devices could also be attributed to interobserver variability. In addition, the amount of IOP reduction in this study was small, so those differences would be likely to occur. The discrepancy in these results was considered a limitation of our study. As previously reported, our ICare IOP values obtained in the lateral decubitus position were significantly higher than those in the sitting position [1,2,4]. The difference in the IOP between the sitting and lateral decubitus positions (mean of 3.2 mmHg) was similar to a previous study [13]. Additionally, previous reports suggested that trabeculectomy reduces the degree of posture-induced IOP changes [11,12,13]. In this study, iStent inject W did not reduce the degree of posture-induced IOP changes. One explanation for this inconsistency of posture-induced IOP changes may involve the difference in surgical mechanism between trabeculectomy and iStent inject W. The posture-induced IOP changes are caused by choroidal vascular congestion and increased episcleral venous pressure during decubitus position [2]. Trabeculectomy makes a newly aqueous pathway through the filtering bleb independent of the episcleral veins. Reasonably, trabeculectomy suppresses posture-induced IOP variations. Conversely, iStent inject W creates two bypasses that facilitate aqueous outflow through the trabecular meshwork into Schlemm’s canal, and its postoperative IOP is related to the episcleral venous pressure. Differences in these IOP-lowering mechanisms between surgical procedures may lead to inconsistent results. Another explanation is the difference in the amount of IOP reduction between trabeculectomy and iStent inject W. The IOP reduction effect of MIGS procedures is lesser than trabeculectomy [42]. Preoperative IOP is typically lower in cases where iStent inject W is indicated than that of trabeculectomy. Therefore, the positive effect of iStent inject W on posture-induced IOP changes may be difficult. Other MIGS, such as Kahook dual-blade (New World Medical, Rancho Cucamonga, CA, USA), have a higher IOP reduction than iStent surgery series [43,44,45]; thus, other MIGS procedures may reduce the degree of posture-induced IOP changes. A further evaluation by other MIGS procedures would be required in the future to dispel this concern.

Previous reports suggested that cataract extraction is an effective surgery to lower IOP in patients with glaucoma. IOP lowering is more significant in eyes with narrow angles and those with higher baseline IOP levels, whereas eyes with IOP in the lower range of normal tend to have an IOP that is unchanged from baseline or even higher following cataract extraction [46,47]. In this study, the patient’s eyes had open angles and normal ranges of preoperative IOP; therefore, the lowering IOP effect of cataract extraction may have been low. However, we could not evaluate the possibility of the effect of cataract extraction on posture-induced IOP change, which is also a limitation of this study.

Postoperative complications, such as hyphema and IOP spikes, were not observed in any cases. Two eyes (13.3%) experienced one stent occlusion by the iris. The stent occlusion has not required the resolution using the YAG laser because of the enough IOP reduction in this study, although the occlusion can be resolved with the YAG laser [48]. Secondary glaucoma surgery for IOP control was not required in any case. The iStent inject W procedure may not adversely affect the corneal endothelial cells [22,48,49] because corneal ECD reduction (2.3%) in this study is similar to or less than the range expected after cataract surgery alone [50]. The visual acuity was stable or improved through 12 months postoperatively, indicating that the iStent inject procedure did not detract from the visual improvements expected after cataract surgery. The visual fields remained stable or improved during the study. The improvement in visual fields postoperatively may be due to cataract surgery. The frequency of these postoperative complications and the improvements in visual acuity and visual fields are consistent with previous studies [23,26,27,28,48]. These findings suggested that iStent Inject W with phacoemulsification has excellent safety, easy postoperative management, and disease stability effect.

We are aware of the limitations of our study. First, the sample size was small, and the follow-up period was short. The effect of IOP reduction by iStent inject W may not be accurately assessed. Therefore, our results may be preliminary. Future research should be done over longer periods of data collection, as well as with patients from multiple sites and/or with larger sample sizes, to resolve this limitation. Second, there was no control group of patients receiving phacoemulsification alone, which would determine whether iStent is effective in the posture-induced IOP changes and controlling visual field impairment. Third, we did not evaluate the posture-induced IOP changes in ocular perfusion [51] and cerebrospinal fluid pressure [52], which may play a compensatory role in IOP changes. Fourth, the results may not accurately reflect important features of the physiological and environmental sleeping status of our participants because this study was performed in the daytime.

## 5. Conclusions

iStent inject W combined with phacoemulsification achieved a reduction in IOP and the number of glaucoma medications during short-term follow-ups with high safety. However, iStent inject W did not affect the degree of posture-induced IOP changes. Therefore, we should select filtering surgery for cases that want to reduce the degree of posture-induced IOP changes.

## Figures and Tables

**Table 1:** Patient characteristics.

Characteristics	Total (*n* = 15)
Age (years)	73.1 ± 6.3
Sex, *n* (%)	
Male	4 (27)
Female	11 (73)
Glaucoma type, *n* (%)	
Primary open-angle glaucoma	12 (80)
Exfoliation glaucoma	3 (20)
IOP with GAT (mmHg)	16.0 ± 2.6
Number of glaucoma medications, *n*	2.5 ± 1.2
BCVA (logMAR)	0.45 ± 0.3
Central corneal ECD (cell/mm^2^)	2525 ± 321
Visual field MD (dB)	−9.8 ± 4.6

Data are shown as the mean ± standard deviation. BCVA, best corrected visual acuity; GAT, Goldmann applanation tonometer; IOP, intraocular pressure; logMAR, the logarithm of the minimum angle of resolution; *n*, number; MD, mean deviation.

**Table 2:** Postoperative complications.

Complication	*n* (%)
Hyphema	0 (0)
IOP spikes	0 (0)
One iStent occlusion by iris	2 (13)
Additional glaucoma surgery	0 (0)

IOP, intraocular pressure.

## Data Availability

Data is fully available upon reasonable request to the corresponding author.
